# Owner experience and veterinary involvement with unlicensed GS-441524 treatment of feline infectious peritonitis: a prospective cohort study

**DOI:** 10.3389/fvets.2024.1377207

**Published:** 2024-06-26

**Authors:** Rosa Negash, Emma Li, Nicole Jacque, Wendy Novicoff, Samantha J. M. Evans

**Affiliations:** ^1^Department of Veterinary Biosciences, College of Veterinary Medicine, The Ohio State University, Columbus, OH, United States; ^2^Independent Researcher, San Jose, CA, United States; ^3^Department of Orthopedic Surgery and Public Health Sciences, School of Medicine, University of Virginia, Charlottesville, VA, United States; ^4^Department of Microbiology, Immunology, and Pathology, Colorado State University, Fort Collins, CO, United States

**Keywords:** anti-viral therapy, coronavirus, citizen medicine, human–animal bond, cat

## Abstract

**Introduction:**

Feline Infectious Peritonitis (FIP) has historically been a fatal coronavirus disease in cats. In recent years, the therapeutic agent GS-441524, developed by Gilead Sciences, was found to be a successful treatment for FIP in most patients in clinical trials. However, this particular drug has remained stalled in the therapeutic pipeline, leaving patients and cat owners without a licensed medication. In the meantime, online social media platforms began to emerge, connecting cat owners with a community of citizen non-veterinary professionals sourcing unlicensed GS-441524.

**Methods:**

This study prospectively followed participants (*N* = 141) that successfully completed 12 weeks of treatment, capturing their treatment experiences with self-administered GS-441524-like medication. A one-time survey was administered to enrolled participants with mixed format of questions (open-ended and multiple-choice) asking about treatment administration techniques, observed side effects of GS-441524, accrued cost, veterinarian involvement, impact on the cat-human bond, and social media usage.

**Results:**

Our results show cat owners experienced a shift in treatment modality from injectable GS-441524 to pill formulation across the treatment period. The average total cost of medication has decreased since 2021 to approximately USD 3100, and participants reported the human–animal bond being affected negatively. Additionally, there was an increased trend in veterinarian awareness of GS-441524-like therapeutics and monitoring of clients undergoing treatment. Social media usage was reported as being important at the beginning of treatment to establish treatment administration but lessened by the end of treatment.

**Discussion:**

This study is the first detailed, prospective account of owner experiences with unlicensed GS-441524, raising an important discussion surrounding citizen veterinary medicine.

## 1 Introduction

Feline Infectious Peritonitis (FIP) has been a devastating disease to encounter in the veterinary health field for decades. FIP is caused by the first of two pathotypes of feline coronavirus (FCoV)—feline infectious peritonitis virus (FIPV) and feline enteric coronavirus (FECV)-- and is characterized by a rapid onset of signs which can include abdominal swelling, uveitis, and neurological signs (1). However, presentation of these signs alone does not indicate disease and diagnostic testing for FIP is challenging as the virus is difficult to distinguish from it’s relatively non-pathogenic counterpart (FECV) (2–4). Until recently, FIP was considered fatal in cats, with most cases being diagnosed during necropsy (5). An experimental treatment called GS-441524, which is a nucleoside analog antiviral drug, was developed by Gilead Sciences and has shown promise as an effective therapy for FIP (6). Unfortunately, the development of this treatment was halted, presumably to prioritize efforts in creating a therapeutic for RNA viruses affecting humans (7).

Critics of the unlicensed use of GS-441524 have urged for the use of alternative therapeutics such as Molnupiravir and Remdesivir as a first-line therapeutic in treating FIP. However, until very recently, neither drug was available for off-label prescription use by a veterinarian, with approval still pending for Molnupiravir (8). Moreover, even with a prescription, both are estimated to be considerably more expensive than unlicensed GS-441524 (9, 10). Molnupiravir has been found to be an effective rescue therapy after failed use of GS-441524, but so far is not often used as a first-line therapeutic in treating FIP (6, 10).

In 2020, Remdesivir received full FDA approval for treatment of COVID-19 in adult people (11); however, some public health officials and researchers have called for FDA approval of GS-441524 instead, for on-label use to treat COVID-19 in humans. Preliminary research suggests GS-441524 as a potentially more viable antiviral therapeutic than Remdesivir in treating COVID-19 in humans, citing a simpler chemical structure and improved *in vivo* efficacy in veterinary studies, primarily in cats (12). In response to this growing demand, several online social media platforms emerged that connected owners with resources to acquire unlicensed GS-441524 products and provided a social network to discuss treatment techniques (13). Yet, these platforms had very little oversight from veterinarian practice or regulatory associations, leaving a gap in care and involvement from licensed professionals. We have recently analyzed the GS-441524 and remdesivir content, as well as the pH, of some of the most widely-available black market products, and found them all to contain GS-441524 (14).

There is limited knowledge on veterinarian involvement and management of feline patients undergoing unlicensed GS-441524 therapy for FIP. As part of a larger research effort to understand the efficacy and survivability of cats being treated with unlicensed GS-441524, our research group aimed to examine current trends in veterinary support of unlicensed therapy treatments. We also captured participant experience with administering GS therapy and their likelihood to use GS therapy in the future. We hypothesized that participants had improved veterinarian support, but still relied most heavily on social media engagement with non-licensed ‘citizen’ guidance.

## 2 Materials and methods

### 2.1 Study design and participant recruitment

We prospectively collected survey data using a convenience sample of participants engaged in GS-441524 therapy. Our sample frame was members of a prominent social media platform, primarily found on Meta’s Facebook platform, that lived predominantly in the United States. Following institutional review board (protocol #2021E0162) approval from our institution, we designed 6 surveys capturing information on GS-441524 therapy efficacy, survival rate, dosing, treatment completion, complications, relapsing, and veterinary support. Using survey templates from the retrospective study, we developed our survey questions to have a 6th-grade lexicon level of readability and used conditional logic to only populate questions if they were applicable to the previously answered question(s). Beta testing of surveys was completed with a focus group of participants and their feedback was incorporated into the final version of the surveys. Surveys were designed using Qualtrics XM software (Qualtrics, Provo, UT, version January 2022).

To recruit participants, we created a study flyer advertising our research study and a link to our pre-enrollment survey. We communicated with several administrators and moderators working on the FIP Warriors platform and requested permission to share the study recruitment flyer on the FIP Warriors main Facebook page. Participants were also recruited into the study by word-of-mouth communication and frequent Facebook posts by moderators which advertised our study and provided contact information. We broadly posted the study flyer a total of 3 times throughout the study window and shared study recruitment information online and at several conferences. There was no monetary incentive, but we provided a unique key chain to each participation as an incentive to commemorate their participation in our study. Importantly, all participants were recruited at diagnosis or the initiation of therapy to prevent bias toward positive outcomes.

The study enrollment window was from January 01, 2022 to July 1st 2022. Participants who responded to study recruitment messaging received a link to the pre-enrollment survey. This first survey asked for participant name and email. Once completed, study personnel downloaded and stored participant contact information in a secure, master list. Each participant received a study identification number to link all survey responses; if a participant had multiple cats undergoing GS-therapy, a unique identification number was issued for each cat. Within 48 h, participants received a link to the enrollment survey, which asked a series of in-depth questions related to their cat’s signalment, diagnosis of FIP, clinical signs, GS-441524 dosing, and other GS-related information. Participants who completed the enrollment survey progressed to the treatment phase of surveys, which included a weekly survey of questions documenting the therapeutic regimen and any changes in their cat’s behavior, physical appearance, and dose–response to GS-441524. Participants received automated emails once a week for a minimum of 12 weeks to record and observe weekly changes for patients undergoing GS therapy.

To track participant responsiveness and survey completion, we created a participant tracker key, which designated participants’ level of survey engagement and GS-therapy outcome status. Throughout the survey study window, we recorded the percentage of survey completion for each participant as ‘Full,’ ‘Partial,’ ‘Initial Only,’ or ‘None’ (descriptions can be found in Supplementary Table 1). Because our surveys were released in treatment-phase intervals, we prospectively evaluated survival outcomes based on survey responsiveness and completeness. Every week, we sent out an initial batch email to all active participants. After 48 h, a reminder email was automatically sent out to participants that had not completed the weekly survey. Study personnel would individually follow up with participants if they had missed more than 3 weeks in a row of survey reminders.

### 2.2 Defining treatment completion

During the final week (Week 12) of weekly surveys, participants were asked to mark if they were finishing GS-therapy treatment that week or continuing. If participants selected ‘Yes,’ they were marked as complete and progressed into the Observation phase of surveys. If participants selected ‘No,’ participants were tracked until they indicated they had finished treatment (marked as ‘extended treatment’) or if their cat died at any point during treatment (marked as ‘premature death’). Study personnel followed up with participants at least twice to determine their treatment status; only participants that indicated they had completed 12+ weeks of treatment were then sent the treatment completion survey. A treatment completion survey was sent out during Week 13, which asked participants questions on treatment completion and satisfaction, veterinary support during treatment, as well as direct and associated costs of GS-therapy (Supplementary material 1). The purpose of this survey was to capture information on the participants’ experience with GS administration to treat FIP as well as how they navigated both online/social media citizen medicine platforms and traditional veterinary medical care.

### 2.3 Analysis

This survey consisted of 27 questions with a mix of multiple choice and write-in short answer formats. Descriptive statistics were computed to estimate average costs of GS-441524 like products, with standard deviation. Count (N) and proportions (%) were tabulated for multiple choice responses. Likert scales were reported of questions using 5-point rating scales. Using open and *in vivo* coding methods, qualitative responses were analyzed to examine participant attitudes, beliefs, and experiences. Analysis was performed in Excel (version 16, Microsoft Corporation).

## 3 Results

This survey included 143/151 responses (94.7%) from the participants that fully completed the weekly treatment stage (weeks 1–12+). The average total cost of GS-441524 medication cost was 3,103 USD (STDV: 3103.1), with a range of 0–10,000 USD. Participants spent an additional 2,437 USD (STDV: 3270.7) on veterinary monitoring (bloodwork, physical exams) and on supplemental therapies (ex: supportive care or medications such as gabapentin or B12 supplementation), with a range of 0–20,000 USD. In addition, participants reported an average of 129 USD (range 0–1,250 USD) on ‘wasted’ GS-441524 products due to improper storage, injection material that did not make it into the cat, etc.

### 3.1 Establishing a GS-441524 routine

**Figure 1:**
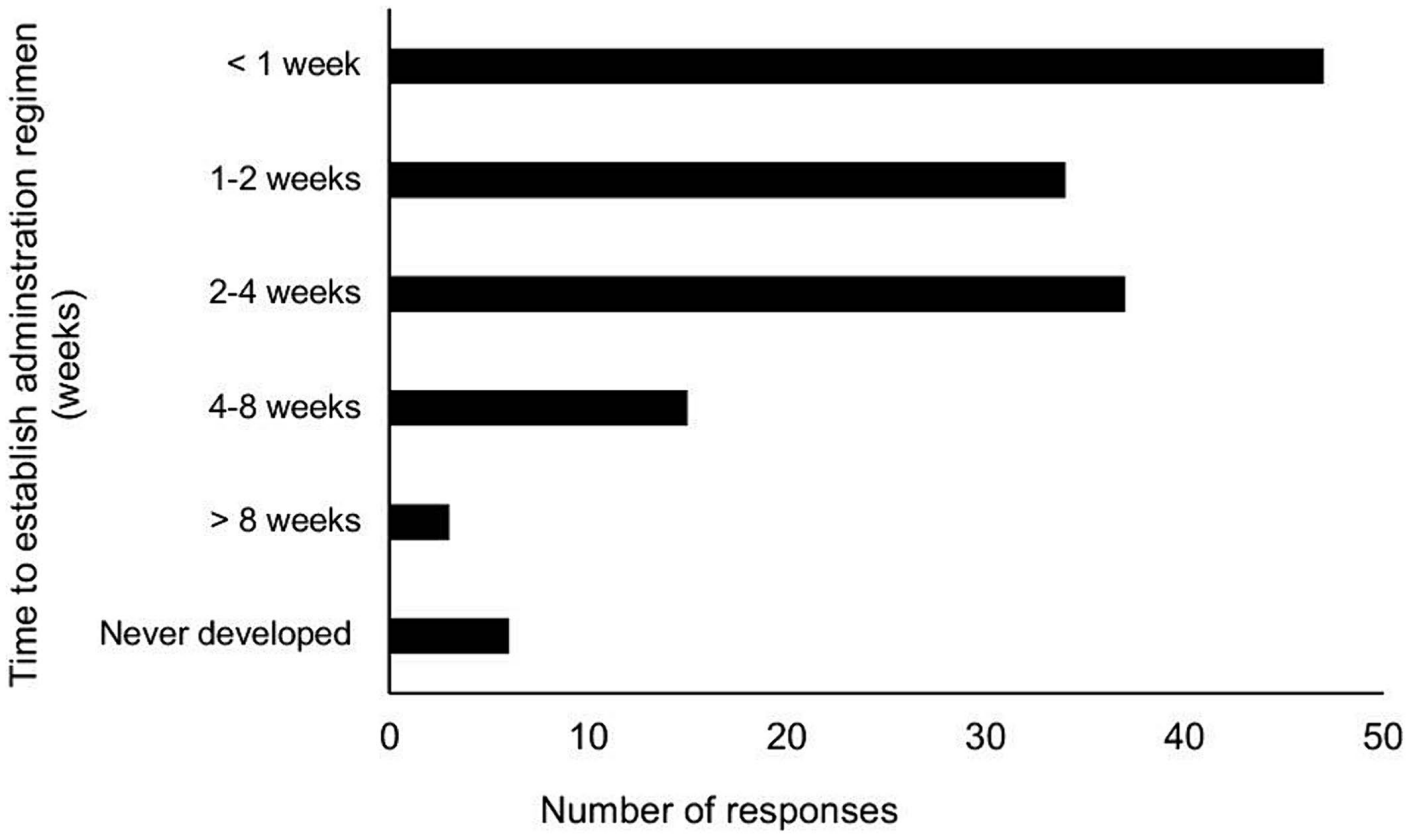
Amount of time to establish a GS-441524-like administration regimen among participants undergoing 12-week treatment cycle, using either injectable or oral GS-441524-like product.

Regarding the establishment of a treatment administration regimen and the perceived difficulty of delivering GS-441524-like medication, most participants accomplished this within 0–2 weeks (57%), followed by 26.1% within 2–4 weeks (Figure 1). Overall, the majority of participants found it difficult or moderately difficult to administer the correct dose and formulation of GS-441524-like therapy throughout the treatment period (Table 1). Furthermore, when rating the daily administration of GS in injectable or oral forms, a lower proportion of participants reported it as difficult or moderately difficult (44%).

**Table 1:** Likert scale gauging participant experience with administering GS-441524-like medication.

Question	Difficult	Moderately difficult	Neither	Moderately easy	Easy
How would you rate the overall difficulty of acquiring the required amount and correct formulation of GS throughout the duration of treatment?	56 (39.4%)	56 (39.4%)	13 (9.2%)	14 (9.9%)	3 (2.1%)
How would you rate the overall difficulty of daily administration of GS (giving injections or oral pills) to your cat?	16 (11.3%)	55 (33.7%)	20 (14.1%)	38 (26.8%)	13 (9.2%)

Participants were asked about the physical signs that they attributed to GS-441524 therapy (Supplementary Table 2). The majority of participants reported their cat as struggling or not being compliant during injections (71%), Vocalizing during injection (69%), increased activity level (65%), experiencing injection site pain (58%), wound at injection site (55%), and increased appetite during treatment (54%). Less common attributes included diarrhea (15%), vomiting (5%), and injection site infection (4%).

A free-response question was provided for participants to further explain their experiences with administering GS-441524 therapy. Participants were asked to optionally share any additional information with the research team on the experience of using GS-441524 therapy to treat FIP in their cats in a free-text response box. This question in the survey was designed to capture information on the direct impact that using GS-441524 like therapy had on cat owners, their relationships with their cats, and the unique difficulties of using a novel, unlicensed therapy outside of the traditional veterinary setting. Qualitative coding of responses resulted in 5 major themes (Table 2).

**Table 2:** Major themes of establishing a GS routine from participant experiences of using GS-441524 like therapy after completing treatment (optional, free-response question).

Theme	*N* (%)^‡^	Description
Difficulty with establishing GS-441524 routine/injecting GS-441524	57/106 (54%)	Lack of training on how to give “vaccine”/injection; cat vocalizing pain when injecting GS; injection site sores, scar tissue; leaking/wasted doses
Requiring help	18/106 (17%)	Participants reported requiring more than one person to administer an injectable dose
Cat-owner dynamic	27/106 (25%)	Cats becoming combative toward owner around the time of administration; cat hiding before injection; Cat jerking or attempting to jump during injection; cats biting or scratching cat owner during administration
Stress/Anxiety of cat owner	19/106 (18%)	Feelings of worry, stress, fear, and guilt surrounding hurting their cat during treatment
Switching to oral formulation	43/106 (41%)	Many participants reported switching to oral formulation of GS during the treatment period and owner relationship greatly improving after switch

^‡^Percentages may add up to more than 100%, as participants could report more than one category in their answers.

Participants provided a range of responses about establishing a GS-441524 therapy routine with their cats. For many participants, the first few weeks of treatment were difficult to navigate because of their lack of medical training in administering injections (often mistakenly referred to as “vaccines” by participants) and the severity of disease in their cats. Participants also mentioned requiring an additional person to be present to hold the cat while the other person administered the medication. Techniques such as swaddling or “burrito-ing” were commonly practiced to restrain the cat to limit injury to both the cat and the owner when injecting GS-441524.

When explaining how they administered a dose, participants mentioned phrases such as ‘tenting’-- pulling the skin upwards to inject the dose subcutaneously– and ‘rotating sites’ to switch the location of injection on the cat. Sores, skin irritation, and scar tissue were listed as common physical side effects of the GS-441524 injection site. Likely due to GS-441524’s highly acidic formulation, cats were also reported to vocalize (howling or yelping) during injection. Several owners reported stress and anxiety before administering GS-44154 injections, reporting themes of sadness, guilt, and fear of hurting their cats. One participant’s comment is found below.

*“It was difficult to tent the skin and know how far to go into the skin since we are not formally trained in this. It was also extremely difficult to keep the cat from jumping away during the injection … At times the injection site would leave a bubble which was worrisome because we weren’t sure if it would absorb correctly. Shaving the fur for the injection sites every 2 weeks seemed to help just to visualize and hold the tent better but not with the actual injection.”*

Many participants also reported their cats becoming familiar with the medication routine and hiding from participants when it was time for their next dose. Strategies to prepare cats for injections included sectioning off parts of the house to limit cats’ hiding places, offering high-value treats such as “Churu” before injection, injecting at the same times each day, and soothing cats with stroking and petting after injection. As their cats began to gain weight during treatment, however, these strategies proved to be less useful as cats were stronger and could physically resist treatment. Participants also stressed the emotional toll this experience had on them, with one participant mentioning:

*“It was definitely a 2 person job, and it became more difficult as the kitten grew! She had so many injection site sores that finding a “safe” spot was extremely hard. We made her bleed on several occasions. This was massively stressful on all of us and heartbreaking to see her face when we picked her up every day at “that time.” We persevered, but hope no one has to go through this again. Ever. We would do it, but the stress has its toll”*

Some participants mentioned that behavior strategies only were not enough to calm down their cat before GS-441524 administration and therefore sought out therapeutic sedative options. Gabapentin was the most commonly reported sedative used to manage anxiety among the cats in the study. However, some participants mentioned that even with the addition of Gabapentin, their cat still struggled with injection delivery of GS-441524.

*“[Redacted] developed sores and very tough skin. Lots of leakage after shots no matter what trick I used. She was in lots of pain with sores near the end of treatment and no matter how much gabapentin I gave her. She was never comfortable.”*

### 3.2 Injection vs. oral formulation

Several participants (41%) stated they began with injectable GS-441524 therapy and switched to oral formulation in the later stages of the 12 week GS-441524 therapy treatment window. Reasons for switching to oral formulation included cat aggression and avoidance (hiding) around administration time, lack of skin sites available to inject into, wasted liquid doses, and owner emotional burnout. Participants reported that their cats become more combative over the course of treatment, which included scratching and biting owners. Cats would also move rapidly and jerk during the injection process, requiring the cat owner to re-inject and the cat having another injection site injury. This was especially difficult for owners because of their uncertainty surrounding whether a full dose was administered and the need to purchase more GS-441524 because of wasted doses. Several participants reported feeling emotionally conflicted with continuing injections of GS-441524 therapy when they discovered oral/‘pill’ formulation of GS-441524. Oral formulation was reported as being significantly easier on both the cat and owner to establish a routine. Information on how they found out about the oral form of GS-441524 was not captured in the survey.

*“We had to change course about 1/3 of the way into treatment. Injections were proving too difficult as our cat was becoming distant and negatively reacting to the administration of the injections. Once we switched to pills, giving the medicine was a dream. It made life much more pleasant for all of us.”*

Cost of oral formulation was mentioned as being higher than injectable GS-441524, with participants having to spend more toward the end of treatment. Oral formulation was rated as being much easier than injectable GS-441524, with no participants reporting difficulty with administering pill form of GS-441524. Overall, participant experience with establishing a therapeutic routine varied across the course of treatment depending on the type of GS-441524 formulation.

**Figure 2:**
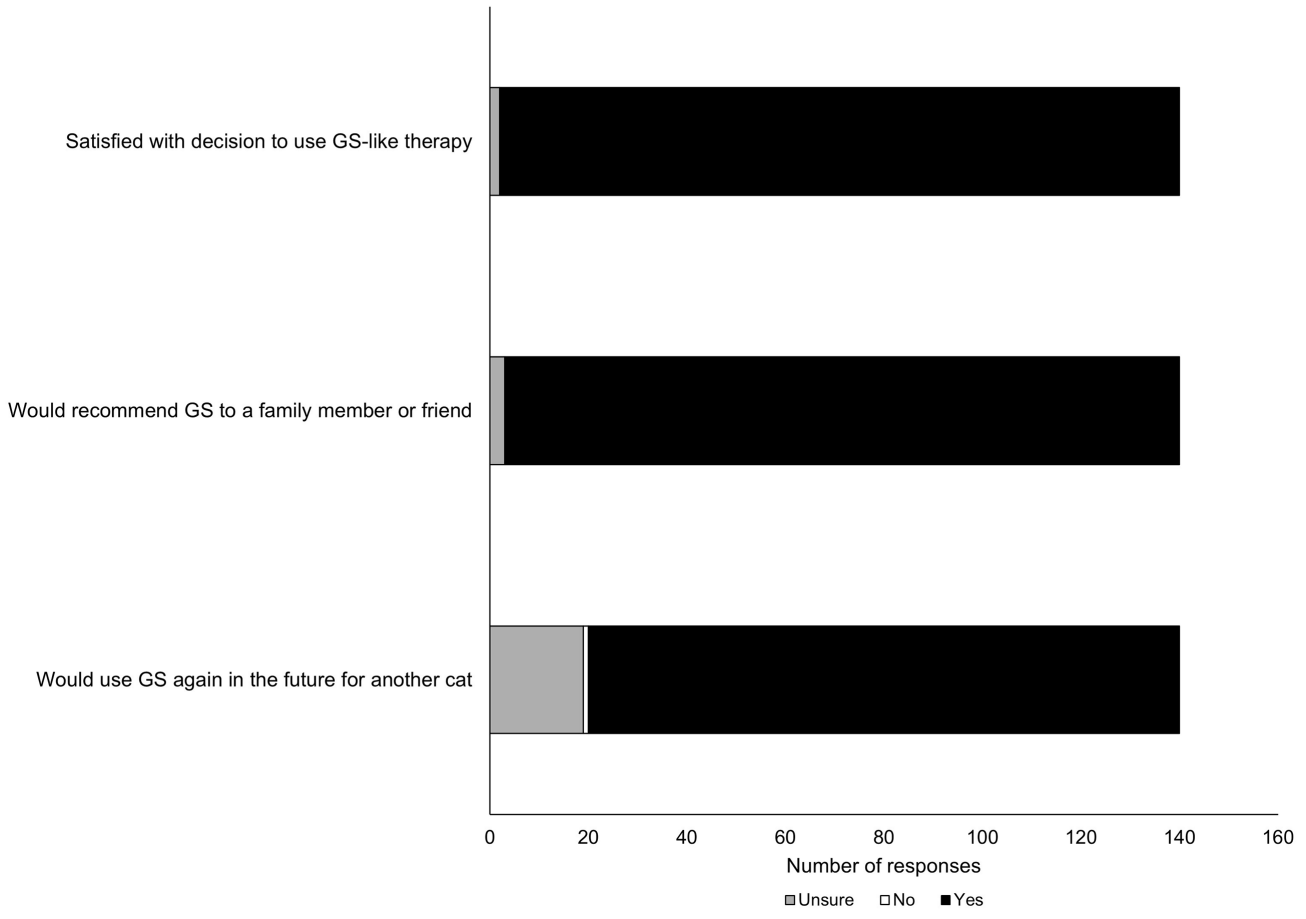
Summary of likelihood of participants’ responses to question posed about future use of GS-441524 like therapy.

It was of interest to this study to gauge participants’ future interest or likelihood of using GS-441524 therapy again in the event their cat was diagnosed with FIP for a second time (Figure 2). The overwhelming majority of participants (98.5%) reported satisfaction with the decision to use GS-like therapy; 98% reported they were likely to recommend GS-441524 like therapy to a family member or friend. However, there was a mixed response from respondents when asked directly if they would use GS-441524-like therapy in the future for another cat, with somewhat fewer people reporting ‘yes’ (86.5%) and more people reporting ‘unsure’ (12.8%).

### 3.3 Veterinary involvement

Assessing veterinary involvement was another key component of this study. 52% (*N* = 73) of participants reported being extremely satisfied with the amount of veterinary assistance they received during the time of treatment. 17% (*N* = 24) reported being somewhat satisfied, 14% (*N* = 21) reported feeling neither satisfied nor dissatisfied, 12% (*N* = 17) replied with somewhat dissatisfied, and lastly, 4% (*N* = 6) reported being extremely dissatisfied.

Participants had mixed responses of the attitude or beliefs of their veterinarians of the participant’s decision to use GS-441524 like therapy to treat their cat’s FIP (Table 3). Two main attitudes were reported: hesitant to help and supportive. Participants provided free response answers describing the context of and potential motivations behind their veterinarian’s attitudes and beliefs.

**Table 3:** Participants’ experiences with and perceived support from veterinarian clinicians and professionals during GS-441524 like treatment (optional, free-response question).

Theme	*N* (%)^‡^	Description
Switched to another veterinarian during treatment/sought out second opinion	17/87 (20%)	Cat owner was either refused service or felt unsupported by primary veterinarian; Cat owner sought out another veterinarian who worked at specialty hospital, had previous patient experience with FIP, from referral, or found second vet from online sources; cat owners reporting higher satisfaction with treatment following second opinion
Veterinarian hesitancy to help	8/87 (9%)	Concerns with legality around supporting patients undergoing GS-441524 like treatment; provided laboratory services but no referral to or unaware of online resources
Received support from Primary Veterinarian	48/87 (55%)	Primary veterinarian provided range of diagnostic services, labs/bloodwork ordering and monitoring, or prescribing supplemental medications (ex gabapentin); cat owner felt emotionally supported in decision to use GS-441524 like therapy by vet; veterinarian did not administer or purchase GS-441524 like medication
Received FIP education/resources from veterinarian	17/87 (20%)	Vet provided education on FIP; vet directed cat owner to online resources/platforms
Difficulty in receiving FIP diagnosis	6/87 (7%)	Reporting of issues with validating a diagnosis through histopathology tests; recommendation of euthanasia

^‡^Percentages may add up to more than 100%, as participants could report more than one category in their answers.

Some veterinarian providers were unfamiliar with GS-441524 like treatment prior to the participant undergoing treatment. Providers that were hesitant to help reported serious concern with being affiliated with or appearing to endorse the usage of unlicensed GS-441524 due to the lack of legal sources at the time of the study. Participants reported that their veterinarians expressed the concern of violating licensure requirements by supporting these practices. However, providers were still willing to provide laboratory services (weight monitoring, bloodwork, other diagnostic monitoring) or help the participant seek out other providers for a second opinion or services. Participants reported some veterinarians changing their beliefs about GS-441524 like treatment throughout the course of treatment, as they appeared to be interested in the progress of the cat’s improvement.

*“One of the vets at my clinic said there was no hope, but another vet came in & agreed to monitor bloodwork. They didn't know that much about the meds so they weren't very helpful, but they were willing to learn & have been very supportive of our experience & have referred other patients to the warriors group, so I'm grateful they are helping others save their pets.”*

*“My vet had not previously seen a cat treated for FIP, so they were amazed when he came in for his first visit, post-start of treatment, and saw the vast improvement: all the fluid from his lungs was gone, he'd gained weight, and was noticeably active. All positives. I believe it was illuminating for them, though, in my monthly visits, certain technicians were suspect. The vets I met with to do follow ups were all hopeful this therapy could, one day, be approved for use to treat FIP. I believe, if met with a similar case in the future, the vets I saw would recommend the treatment on the down-low.”*

*“Our veterinarian seems reserved at this time to comment but, is happy to see the cat looking and acting so well.”*

For some participants, their veterinarian had previous experience with managing cases of patients undergoing GS-441524 like therapy. These participants reported feeling very supported and encouraged by their veterinarian. They also expressed their veterinarian being either involved with the online platform ‘FIP Veterinarians Education,’ which was a designated online community network of U.S. based providers to support, share, and learn about new research and clinical experiences with cats undergoing GS-441524 like treatment. Some veterinarians were knowledgeable about FIP treatment options because they had read or attended talks by Dr. Niels Pedersen or others.

*“Our vet has treated other cats with FIP before so she was very knowledgeable and supportive. She directed us to the Facebook group and walked us through the process. She was very happy to hear that [redacted] finished treatment and graduated to observation.”*

**Figure 3:**
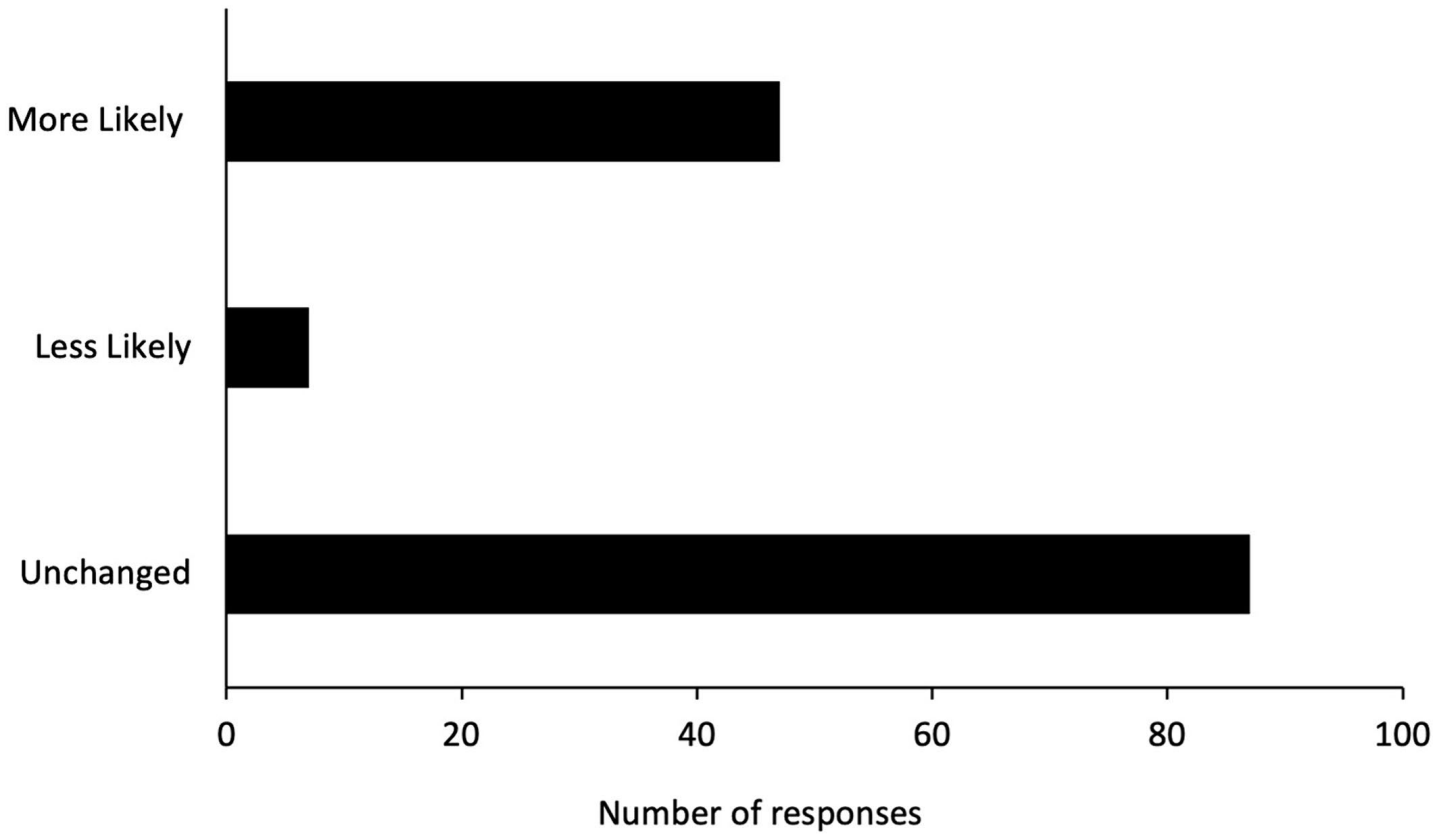
Summary of participants’ responses to whether the experience of unlicensed GS-441524 therapy has made them more, less, or equally likely to seek out future veterinary care.

Participants were asked whether the experience they had with FIP and unlicensed GS-441524 therapy would change their willingness or likelihood to seek veterinary care in the future. 33% (*N* = 47) responded that this experience makes them more willing/likely to seek future veterinary care, 5% (*N* = 7) responded that this experience makes them less willing/likely, and 62% (*N* = 87) responded that this experience has not impacted their willingness to seek future veterinary care (Figure 3).

### 3.4 Social media involvement

In assessing the significance of one-on-one online support during FIP treatment with GS-441524-like treatment, 59 participants (41.8%) regarded it as ‘Extremely Important,’ while 35 (24.8%) considered it ‘Very Important’ (Table 4). Only 3 participants (2.1%) ranked it as ‘not at all important.’ Conversely, responses for Q26, which explored general social media usage and engagement, exhibited a more balanced distribution. ‘Extremely important’ received 41 responses (29.1%), followed by 27 (19.1%) ranking it as ‘Very Important,’ and 20 (14.2%) finding it ‘Moderately Important.’ Lastly, 27.7% marked either ‘not at all important’ or ‘slightly important.’

**Table 4:** Likert scale gauging general social media support from the social media platform ‘FIP Warriors’ Facebook group.

Question	Not at all important	Slightly important	Moderately important	Very important	Extremely important	N/A: I never joined or utilized	Total
How important to your cat’s health and recovery, as well as to your own well-being, was the one-on-one support you received from your social media (ex: FIP Warriors) moderator?	3 (2.1%)	15 (10.6%)	19 (13.5%)	35 (24.8%)	59 (41.8%)	10 (7.0%)	141 (100%)
How important to your cat’s health and recovery, as well as to your own well-being, was the support you received from the general social media (ex: FIP Warriors; apart from your moderator) community?	16 (11.4%)	23 (16.3%)	20 (14.2%)	27 (19.1%)	41 (29.1%)	14 (9.9%)	141 (100%)

## 4 Discussion

This is the first study to prospectively capture the human–animal bond considerations and owner-veterinarian dynamics of cats undergoing unlicensed GS-441524 therapeutic treatment from owner-reported data. The average cost of treatment in this 2022 study was USD 3103, which is down significantly (~40%) since 2020, where the reported average cost of treatment was USD 4920 (14). The majority of owners reported experiencing a shift in the mode of administration during the treatment period, switching from injectable to oral administration. Participants also cited several contributors of stress and anxiety during the treatment period. As a result, participants reported a high level of engagement with social media platforms to receive education on and support in navigating FIP treatment options using an unlicensed, GS-441524 like therapeutic. Furthermore, the involvement of veterinary medical professionals varied depending on veterinarian level of familiarity with GS-441524 like therapeutic. As a disclaimer, this study does not aim to endorse the use of unlicensed GS-441524 nor comment on individual providers’ decision to support FIP treatment using GS-441524-like therapeutics; rather, we aimed to document the current treatment trends surrounding FIP using unlicensed GS-441524 like therapeutics after the successful completion of treatment.

### 4.1 Administration of medication

Since the development of GS-441524, several iterations of the therapeutic have become available internationally (15, 16). GS-441524 has a low pH solubility of approximately 1.5, creating an acidic compound that is very painful when injected below the subcutis (17). This can lead to wounds and fibrosis (scar tissue) at the site of injection (15). Participants in the study reported sustained difficulty with injecting the GS-441524-like products. We did not collect dosing information in the survey analyzed here, though dosage, clinical aspects, and outcome information from this same cohort will be analyzed in future studies under preparation by our group. Furthermore, we have analyzed commonly-used GS-441524 formulations as part of a separate study (14).

Our data reveal a switchover from injectable to oral GS-441524. Participations cited the emotional burden of daily injections as the main motivation to seek out oral formulation instead. Using their online networks, participants mentioned that they were recommended to begin treatment using the injectable form, but later switched to pills because of the difficulty in administering the medication with injections. We observed a clear shift in the human–animal bond, with many participants reporting relief and ease of treatment administration after switching to oral formulation. Oral medication was reported to be more expensive than injections, even with prices reportedly decreasing (18). These findings add important context of the types of medication clients are using and highlight the importance of continued research into feasible modalities of GS-like therapeutics that limit physical and emotional harm to the patient.

### 4.2 Veterinarian involvement

The vast majority of our participants (97%, data not shown) were from the United States and therefore veterinarians referenced in our study follow AVMA guidelines, certification, and FDA licensing requirements. In 2020, participants reported an estimated 8.7% of veterinarians offered education or support (laboratory monitoring, supplemental medications) during the course of treatment using unlicensed GS-441524 (15). Our results show a large increase in the level of perceived veterinary support, even though legality surrounding GS-441524 has not changed. This may be because public awareness of FIP treatments has significantly increased since the first reported manufacturing of unlicensed GS-441524. Pedersen et al.’s seminal paper on the efficacy of GS-441524 nucleoside analog provided breakthrough evidence for treatment for suspected FIP. However, Gilead Sciences did not pursue commercial licensing or FDA approval for use in cats; instead, Remdesivir was prioritized as the target drug for approval (19). The advent of the COVID-19 pandemic of the novel SARS-CoV-2 virus, also a coronavirus, induced a momentous effort to find treatment therapeutics, vaccines, and other agents to control the spread of infection (20). However, GS-441524 was not authorized for use in human or veterinary use and clinical trial findings of GS-441524 to treat COVID-19 remain unpublished (21). This spurred the public-at-large to begin seeking out therapeutics through alternative avenues.

Our study highlights the impact of citizen medicine on novel therapeutics in veterinary medicine. Most participants used online platforms to triage their cats’ clinical signs and obtain GS-441524 like medications. Licensed veterinarians (DVM, VMD) face legal restrictions preventing them from endorsing or prescribing unlicensed drugs, limiting their ability to support clients with suspected FIP (22). Participants sought second opinions when their primary provider lacked support, raising concerns about patient oversight and trust. Lack of professional oversight for patients undergoing FIP treatment could potentially impact the health and safety of the affected cats and lead to a growing mistrust between cat owners and practitioners. However, our study also demonstrates that once veterinary providers were engaged in the treatment of patients with FIP, providers educated themselves on the types of treatments available and exercised clinical judgment within the uncertain treatment landscape. Additionally, the variety of vendors supplying GS-441524 like therapeutics poses challenges due to inconsistent quality and lack of regulatory agency verification (14). Surprisingly, only 5% of participants reported decreased likelihood of seeking future veterinary care, likely influenced by changing to more supportive veterinary practitioners. Recently, Stokes Pharmacy and Bova Group partnered to offer U.S.-made GS-441524 like oral treatment for FIP (23). As public awareness and knowledge about GS-441524 like therapeutics continue to grow, the extent of their impact on veterinary healthcare remains unknown.

### 4.3 Social media usage and legal proceedings

In the field of veterinary medicine, citizen involvement in sourcing and distributing unlicensed GS-441524-like medication for treating suspected FIP has grown significantly (15). Online platforms like ‘FIP Warriors’ have reached thousands of members (53,000 at the time of publication) (13). Much of the current product being purchased via ‘FIP Warriors’ is being manufactured by companies such as Mutian, a pharmaceutical company based in China, whose GS-441524 formulation Mutian Xraphconn is not approved for use by the FDA (16, 24–26). This ‘underground’ sourcing lacks regulation, leading to concerns about product validity and price gouging (27, 28). This phenomenon observed in the United States contributes to a growing international body of literature capturing contributes instances in veterinary health sciences where lay citizens are at the forefront of treating a viral disease with a novel therapeutic approach.

### 4.4 Limitations

This nature of survey collection has a few, inherent limitations. First, self-reported information from participants can induce measurement error, including recall bias due to participants needing to report information from across the 12-week study period. However, because participants were asked to document changes in weekly surveys prior to completing the treatment completion survey, we believe the responses captured in this survey had increased accuracy due to consistent documentation, thus reducing this bias. Additionally, many participants reported having personal documentation of treatment experiences in the forms of daily logs and veterinarian visits that they mentioned in the qualitative responses. Other limitations included loss-to-follow up of participants: at least 3 attempts were made to contact participants that did not complete the weekly treatment surveys, yet a small number of participants’ outcomes were unknown. We also captured information indirectly about veterinarian support during treatment. As veterinarians were not surveyed directly, the information and analysis of veterinarian-related data does not reflect the professional stances or experiences of the referenced veterinarians. Finally, a quick note on terminology: the relevant social media platform uses terms such as ‘moderator’ and ‘administrator’ to differentiate between levels of support staff on the website. For the purposes of this survey, we used the term ‘moderator’ to mean the support staff member that provided direct support to participants while their cat underwent treatment for FIP. However, these terms may differ in their interpretation between participants.

## 5 Conclusion

This study provides novel findings of the cat-owner-veterinarian dynamics in treating suspected FIP with unlicensed GS-441524 like medications. Study findings included a favoring of oral formulation over injectable, citing cat’s pain and resistance (hiding, scratching) during injection administration and ease of delivery using oral pills. Additionally, veterinary support during the 12-week treatment period was assessed. Participants expressed general support toward veterinary involvement, with a higher satisfaction reported amongst veterinarians that had received education on FIP or encountered previous patients with FIP. However, this study provides concerning evidence of poor veterinary oversight and care management, likely due to the legal ramifications associated with unlicensed GS-441524. This study highlights the importance of further research into the trends of GS-441524 like medication use to treat suspected FIP, drug content analyses of GS-441524 like medications, as well as the critical importance of veterinary involvement and oversight in monitoring the health of feline patients being treated with GS-441524 like therapeutics.

## Data availability statement

The raw data supporting the conclusions of this article will be made available by the authors, without undue reservation.

## Ethics statement

The studies involving humans were approved by Ohio State University Institutional Review Board (IRB). The studies were conducted in accordance with the local legislation and institutional requirements. The participants provided their written informed consent to participate in this study.

## Author contributions

RN: Conceptualization, Data curation, Formal analysis, Investigation, Methodology, Visualization, Writing – original draft, Writing – review & editing. EL: Conceptualization, Data curation, Investigation, Writing – review & editing. NJ: Conceptualization, Investigation, Methodology, Writing – review & editing. WN: Data curation, Formal analysis, Writing – review & editing. SE: Conceptualization, Funding acquisition, Methodology, Project administration, Supervision, Writing – original draft, Writing – review & editing.
