# Supplementary material for: Owner experience and veterinary involvement with unlicensed GS-441524 treatment of feline infectious peritonitis: a prospective cohort study
Source: Front Vet Sci. 2024 Jun 26;11:1377207. doi: 10.3389/fvets.2024.1377207 (PMC11233523; doi:10.3389/fvets.2024.1377207)
Supplement: Supplementary file 1 [file Table_1.docx]

Supplementary Material

**Owner Experience and Veterinary Involvement with Unlicensed GS-441524 Treatment of Feline Infectious Peritonitis (FIP): A Prospective Cohort Study**

**Rosa Negash^1^, Emma Li^1^, Nicole Jacque^2^, Wendy Novicoff^3^, Samantha J.M. Evans^4*^**

*** Correspondence:** Samantha J.M. Evans: [Samantha.Evans@colostate.edu](mailto:Samantha.Evans@colostate.edu)

# Supplementary Data

Survey Instrument – attached as separate file

# Supplementary Figures and Tables

**Supplementary Table 1**. Description of denotations used to describe participants’ survey completeness. Participants were tracked throughout the survey collection period and denoted after the survey collection period closed based on the number of completed surveys received.

| **Completeness** | **Description** |
| --- | --- |
| Full | Completed Enrollment Survey, and did not miss more than 3 weeks in a row of Surveys Overall |
| Partial | Completed Enrollment Survey but missed 4 or more weekly surveys in a row |
| Initial Only | Completed Enrollment Survey but then became completely unresponsive at some point during treatment, thus, outcome unknown |
| None | Did not complete any surveys after recruitment |

**Supplementary Table 2.** Signs attributed to GS-441524 therapy during the 12-week treatment window. Participants had the option of marking multiple choices as applied.

| **Sign** | **N (%)** |
| --- | --- |
| Struggling/not being compliant during injections | 101/141 (71.1%) |
| Vocalization during or shortly after injection | 98/141 (69.0%) |
| Increased activity level | 92/141 (64.8%) |
| Injection site pain | 82/141 (57.8%) |
| Injection site sore or open wound | 78/141 (54.9%) |
| Increased appetite | 77/141 (54.2%) |
| Injection site bleeding | 49/141 (34.5%) |
| Injection site swelling | 46/141 (32.4%) |
| General behavioral changes not just immediately before and after injections (ie. hiding, unfriendliness, apprehension around people they are normally comfortable with, etc) | 28/141 (19.7%) |
| Diarrhea | 21/141 (14.8%) |
| Vomiting | 7/141 (4.9%) |
| Injection site infection | 6/141 (4.2%) |
| Decreased appetite | 5/141 (3.5%) |
| Decreased activity level | 3/141 (2.1%) |
| Excessive salivation after oral administration of GS medication | 3/141 (2.1%) |
| Other | 15/141 (10.9%) |
| No, my cat did not experience any of these signs attributed to GS therapy | 3/141 (2.1%) |
