# Supplementary material for: Owner experience and veterinary involvement with unlicensed GS-441524 treatment of feline infectious peritonitis: a prospective cohort study
Source: Front Vet Sci. 2024 Jun 26;11:1377207. doi: 10.3389/fvets.2024.1377207 (PMC11233523; doi:10.3389/fvets.2024.1377207)
Supplement: Supplementary file 2 [file Table_2.docx]

FIPWPS - Treatment Completion/Satisfaction Survey - Final

Start of Block: Basic ID

Q1 Do you allow the information you share to be (confidentially) provided for investigations into FIP and FIP treatment? No identifying information about you, your cat, nor your veterinarian will be shared, including your name, your administrator's name, your pet's name, your email address, nor any mention of your veterinarian/vet practice/employees. Only de-identified information in this survey will be used (in aggregate) for the purposes of advancing research into the treatment and clinical monitoring of FIP in cats.

- Yes (1)
- No (2)

Skip To: End of Survey If Do you allow the information you share to be (confidentially) provided for investigations into FI... = No

| Page Break |  |
| --- | --- |

Q3 What is your email address? Please use the same email address that you have been using for our study.

________________________________________________________________

Q5
What is your first name (or nickname/preferred name)? Please use the same name/nickname for the entirety of this study.


Note: This information is collected only to help us correlate your current and past survey responses, and for addressing you appropriately in personal emails should the need arise.

________________________________________________________________

Q7 What is your cat's name?

________________________________________________________________

Q28
Who was your FIP Warriors group admin? Please list only the admin responsible for helping you manage the majority of your cat's treatment if you had more than one.


Please write "N/A" if you did not have an FIP Warriors admin.


Please note: this will be used for sorting purposes only and your admin's name will remain confidential.

________________________________________________________________

End of Block: Basic ID

Start of Block: GS Administration

| 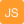 |
| --- |

Q6 What was the date of your cat's last dose of GS?

|  | Month | Day | Year |
| --- | --- | --- | --- |
|  |  |  |  |
| Please Select: (1) | ▼ January (1 ... December (12) | ▼ 1 (1 ... 31 (31) | ▼ 1900 (1 ... 2049 (150) |

Q8 How would you rate the **overall** difficulty of acquiring the required amount and correct formulation of GS throughout the duration of treatment?

- Easy (1)
- Moderately easy (2)
- Neither easy nor difficult (3)
- Moderately difficult (4)
- Difficult (5)

Q9 How would you rate the **overall** difficulty of daily administration of GS (giving injections or oral pills) to your cat?

- Easy (1)
- Moderately easy (2)
- Neither easy nor difficult (3)
- Moderately difficult (4)
- Difficult (5)

Q10 How long did it take you to develop a regular regimen/strategy/method of GS administration that seemed to work well for you and your cat?

- Less than 1 week (1)
- 1 week to 2 weeks (2)
- 2 weeks to 4 weeks (3)
- 4 weeks to 8 weeks (4)
- More than 8 weeks but we did evntually develop a successful dosing strategy/method prior to the end of treatment (5)
- We never developed a dosing strategy/method that worked well for me and my cat; administering GS to my cat was a significant challenge every single day (6)

Q11 (Optional) Please provide any additional comments you have regarding the difficulty of daily administration of GS therapy to your cat:

________________________________________________________________

________________________________________________________________

________________________________________________________________

________________________________________________________________

________________________________________________________________

| Page Break |  |
| --- | --- |

Q12 Did your cat experience any of these signs that was attributed the the GS therapy? (Please check all that apply)

- Increased activity level (1)
- Decreased activity level (2)
- Increased appetite (3)
- Decreased appetite (4)
- Diarrhea (5)
- Excessive salivation after oral administration of GS medication (6)
- General behavioral changes not just immediately before and after injections (ie. hiding, unfriendliness, apprehension around people they are normally comfortable with, etc): (please describe) (7) __________________________________________________
- Injection site pain (8)
- Injection site swelling (9)
- Injection site bleeding (10)
- Injection site sore/open wound (11)
- Injection site infection (12)
- Struggling/not being compliant during injections (13)
- Vocalization during or shortly after injection (14)
- Vomiting (15)
- Other: (please specify) (16) __________________________________________________
- ⊗No, my cat did not experience any of these signs attributed to GS therapy (17)

End of Block: GS Administration

Start of Block: Attitudes/beliefs toward GS therapy overall

Q11 How satisfied are you with your experience of undergoing GS therapy for your cat?

- Extremely dissatisfied (1)
- Somewhat dissatisfied (2)
- Neither satisfied nor dissatisfied (3)
- Somewhat satisfied (4)
- Extremely satisfied (5)

Q12 Now that treatment is complete, given the opportunity to go back in time, would you still have undergone GS-therapy for this cat?

- Yes (1)
- No (2)
- Unsure (3)

Q13 Please elaborate on your answers to the previous two questions.

________________________________________________________________

________________________________________________________________

________________________________________________________________

________________________________________________________________

________________________________________________________________

| Page Break |  |
| --- | --- |

Q18 Would you recommend GS therapy to a friend or family member with a cat suspected of having FIP?

- Yes (1)
- No (2)
- Unsure (3)

Q19 In the future, if you have another cat diagnosed with FIP, would you be willing to undertake GS therapy for them?

- Yes (1)
- No (2)
- Unsure (3)

End of Block: Attitudes/beliefs toward GS therapy overall

Start of Block: Financial burden of GS therapy

| 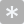 |
| --- |

Q14
What is your best estimate of the total amount of money spent, in US dollars, **on GS medication alone**, for your cat? Please round to the nearest whole number.
 
Please note: only numeric answers will be accepted.

________________________________________________________________

| 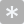 |
| --- |

Q15
What is your best estimate of the total amount of money spent, in US dollars, **on veterinary monitoring and supplemental therapies** undergone during the course of treatment (eg. bloodwork, gabapentin, B12 supplementation, fluid therapy, physical exams, etc.)? Please round to the nearest whole number and do not include any money spent on GS medication.
 
Please note: only numeric answers will be accepted.

________________________________________________________________

| 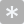 |
| --- |

Q17
How much money would you estimate, in US dollars, was spent on **wasted GS** (ie. injections that did not make it inside the cat, improper storage/mixing causing it to be discarded, etc.)? Please round to the nearest whole number.

 
Please note: only numeric answers will be accepted.

________________________________________________________________

End of Block: Financial burden of GS therapy

Start of Block: Veterinary and community support

Q20 Overall, how much veterinary assistance or involvement did you experience?

- I received virtually no assistance or involvement from any veterinarians (1)
- I received minimal assistance or involvement from my veterinarian(s) (2)
- I received a moderate amount of assistance or involvement from my veterinarian(s) (3)
- I received a good amount of assistance or involvement from my veterinarian(s) (4)
- I received a very significant amount of assistance or involvement from my veterinarian(s) (5)

Q21 How satisfied are you with the amount of veterinary assistance or involvement you received?

- Extremely dissatisfied (1)
- Somewhat dissatisfied (2)
- Neither satisfied nor dissatisfied (3)
- Somewhat satisfied (4)
- Extremely satisfied (5)

Q22 Does the experience you had with the veterinary community throughout the diagnosis of FIP and the treatment of your cat with GS change your willingness or likelihood to seek veterinary care in the future?

- Yes, I feel more likely/willing to seek veterinary care in the future (1)
- Yes, I feel less likely/willing to seek veterinary care in the future (2)
- No, this experience has not impacted my willingness to seek veterinary care for my pets (3)

Q23 (Optional) Please feel free to elaborate on your answers to the previous three questions:

________________________________________________________________

________________________________________________________________

________________________________________________________________

________________________________________________________________

________________________________________________________________

Q24 Please briefly describe the attitude or response of your veterinarian, if any, to your cat's success throughout and after GS therapy: (if you did not have a regular veterinarian or have not spoken with a veterinarian about your cat's improvement of clinical signs, please write "N/A")

________________________________________________________________

________________________________________________________________

________________________________________________________________

________________________________________________________________

________________________________________________________________

| Page Break |  |
| --- | --- |

Q25 How important to your cat's health and recovery, as well as to your own well-being, was the one-on-one support you received from your social media (ex: FIP Warriors) moderator?

- Not at all important (1)
- Slightly important (2)
- Moderately important (3)
- Very important (4)
- Extremely important (5)
- N/A - I never joined or utilized an FIP social media community (6)

Q26 How important to your cat's health and recovery, as well as to your own well-being, was the support you received from the general social media (ex: FIP Warriors; apart from your moderator) community?

- Not at all important (1)
- Slightly important (2)
- Moderately important (3)
- Very important (4)
- Extremely important (5)
- N/A - I never joined or utilized an FIP social media community (6)

| Page Break |  |
| --- | --- |

Q27 (Optional) If you would like to provide any other questions, comments, or details about your cat and your experience that you think might be important to this research, please include them here:

________________________________________________________________

________________________________________________________________

________________________________________________________________

________________________________________________________________

________________________________________________________________

End of Block: Veterinary and community support
